# Investigation of the Defatted Colostrum ^1^H-NMR Metabolomics Profile of Gilts and Multiparous Sows and Its Relationship with Litter Performance

**DOI:** 10.3390/ani10010154

**Published:** 2020-01-16

**Authors:** Diana Luise, Gianfranco Picone, Agnese Balzani, Francesco Capozzi, Micol Bertocchi, Chiara Salvarani, Paolo Bosi, Sandra Edwards, Paolo Trevisi

**Affiliations:** 1 Department of Agricultural and Food Sciences (DISTAL), Alma Mater Studiorum-University of Bologna, Viale Fanin 46, 40127 Bologna, Italy; diana.luise2@unibo.it (D.L.); gianfranco.picone@unibo.it (G.P.); francesco.capozzi@unibo.it (F.C.); micol.bertocchi2@unibo.it (M.B.); chiara.salvarani4@unibo.it (C.S.); paolo.bosi@unibo.it (P.B.); 2 Agriculture-School of Natural and Environmental Sciences, Newcastle University, Newcastle upon Tyne NE1 7RU, UK; agnese.bal@gmail.com (A.B.); sandra.edwards@newcastle.ac.uk (S.E.); 3 School of Veterinary Medicine, Veterinary Sciences Centre, University College Dublin, Belfield, Dublin 4, D04 W6F6, Ireland; 4 Interdepartmental Centre for Industrial (CIRI) Agrofood Research, Alma Mater Studiorum-University of Bologna, Via Quinto Bucci 336, 47521 Cesena, Italy

**Keywords:** colostrum, gilt, IgG, lactose, piglets’ mortality

## Abstract

**Simple Summary:**

Swine colostrum quality and quantity can influence the growth and survival of piglets and contribute to the differences in productive traits of gilts and multiparous sows. The aim of the study was to characterize the soluble metabolomics profile of defatted colostrum of sows at different parity number (PA) and to correlate the metabolomics profile with the colostrum Brix percentage as an estimate measure of immunoglobulin G (IgG) concentration and sow productive traits. The metabolomics profile of colostrum reveals a slight influence of parity, which can influence the quantity of specific metabolites including glycine and lactose. Specific metabolites including lactose creatine, *myo*-inositol, and *O*-phosphocholine partially explain the colostrum IgG Brix percentage. Sows’ productive traits performance, including the litter weight at birth and piglets’ mortality, can be influenced by the metabolites related to a sow’s metabolic condition. Increasing knowledge on the interplay between colostrum composition and litter performance can pave the way to define management strategies to provide piglets with good-quality colostrum, improving welfare and economic sustainability of pig rearing by reducing piglet mortality.

**Abstract:**

The aim of the study was to characterize the soluble metabolomics profile of defatted colostrum of sows at different parity number (PA) and to correlate the metabolomics profile with the Brix percentage estimate of colostrum immunoglobulin G (IgG) and sow productive traits. A total of 96 Meidam (crossbreed Large White × Meishan) sows of PA from 1–4 (PA1: 28; PA2:26; PA3:12; PA4:26) were included, and their productive traits were recorded at 10 days post-farrowing. Colostrum IgG was quantified using a Brix refractometer, and metabolomics profile was assessed using ^1^H-NMR spectroscopy. Sows’ PA slightly influenced the metabolomics profile of colostrum. lactose and glycine were higher in PA1 compared with PA4 (*p* 0.05) and *N*-acetylglucosamine (GlcNAc) tended to be higher in PA2 than PA3 and PA4 (*p* < 0.10). The Brix percentage of IgG was negatively associated with lactose and positively with creatine, *myo*-inositol, and *O*-phosphocholine (*p* < 0.05). Taurine was positively related to litter weight at birth. GlcNAc and *myo*-inositol were linked to piglet mortality at day 10 with a negative and positive trend, respectively. In conclusion, colostrum of gilts and multiparous sows had a similar metabolomics profile. Specific metabolites contributed to explanation of the variability in colostrum Brix percentage estimate of IgG concentration and the sows’ productive performance.

## 1. Introduction

The parity of the sow can be an important factor influencing the growth and survival of piglets. Progeny of first-parity sows can be lighter at birth [1] and later in life [2], and they grow slowly compared with progeny of multiparous sows [1,3]. Gilts are also more prone to farrowing stress and more susceptible to pathogens, and they show a lower capacity to convert energy into milk production compared to multiparous sows [4]. The colostrum quantity and quality may contribute to differences in progeny of first-parity sows compared with multiparous sows. Organic components of colostrum (proteins, fats, and carbohydrates) provide energy to the newborn; furthermore, colostrum is rich in antibodies, immunoglobulins (Igs), leukocytes, selenium, vitamin E, and oligosaccharides which are essential for immune function of the piglets [4,5,6,7] and for the establishment of the microbiota in piglets’ intestine [8], which contribute to intestinal immune system development [9,10].

The effect of parity number on swine colostrum composition was mainly investigated in terms of immunoglobulin, fat, protein, lactose, and net energy concentrations [11,12]. Recently, the development of new technological tools allowed the description of the colostrum and milk metabolomics profile, defined as the comprehensive characterization and quantification of metabolites, which may have a bioactive and beneficial effect on the neonates [13,14]. Few studies investigated the swine colostrum metabolome composition [15,16,17] and the relationship between colostrum metabolites and sows’ productive traits [17,18]. Recently, it was observed that sows’ parity number may influence the colostrum’s lipid composition in terms of free fatty acid (FFA) and de novo fatty acids (FA) related to the energy metabolism (mainly the C18 FA series) [16]. Therefore, it was hypothesized that the colostrum metabolomics profile of different parity sows could differ due to the different degree of maturation and energy conversions of the sows, and this may help to explain the sows’ productive traits. For the purpose of this study, production traits included the sow’s number of piglets (born alive, stillborn), their weight, and their survival to 10 days of age. These production variables indicate the economic performance of a sow during its stay in the herd, and they are important reference points when making breeding and culling decisions.

Therefore, the aims of the present study were (i) to characterize the metabolomics profile of swine colostrum of different parity sows (from 1–4), (ii) to correlate the metabolomics profile with the Brix percentage estimate of colostrum immunoglobulin G (IgG) concentration, and (iii) to evaluate the association between the colostrum metabolomics profile and sow productive traits.

## 2. Materials and Methods

The Secretary of State (UK) authorized Prof. S.A. Edwards of Newcastle University to perform the project (project license number PPL60/4164).

### 2.1. Animals and Sampling

Data were collected from 96 Meidam (crossbreed Large White × Meishan) sows of different parity number (PA)—28 sows in parity one (PA1), 28 sows in parity two (PA2), 12 sows in parity three (PA3), and 26 sows in parity four (PA4)—in a timeframe of four months during the autumn/winter season. Sows were housed in the same breeding facility and were inseminated twice within 24 h by artificial insemination using pooled semen from Meidam boars. During gestation, sows were housed in mixed parity groups (50 sows per group mixed with additional sows not included in the study) and fed by electronic feeding stations twice a day to provide on average 2.5 kg of the same gestation diet (Table S1, Supplementary Materials). At 110 days after insemination, sows were moved from group gestation barns to individual farrowing crate pens with dimensions of 1.83 m × 2.74 m and equipped with a feeder and drinker. Ambient temperature in farrowing rooms averaged 21 °C, and each pen was provided with an infrared lamp for piglets. Sows were fed twice a day until farrowing with the same gestation diet and had free access to water throughout. Sows were not treated with antibiotics or medical products during the gestation and lactation. At the end of farrowing, litter size at birth was recorded, including the total number of piglets born (littersizeTB0), the number of piglets born alive (littersizeBA0), and the number of stillborn piglets (stillborn). Litter weight at birth (LitterWe0) was recorded excluding the stillborn. Cross-fostering was carried out from the second day of life up until weaning. Piglets were initially moved to even up litter sizes and match to the number of functional teats, but subsequently to move poorly thriving piglets to a less competitive environment for welfare reasons. Cross-fostering occurred within parities according to the availability of a suitable foster litter. During the first 10 days post-farrowing, the number of dead piglets in each litter was recorded (total number of dead piglets per nursing sow; livedead). Litter size (littersizeBA10) and weight (litterWe10) at 10 days post-farrowing were recorded, and these values were adjusted for fostering (plus number/weight of fostered piglets out or minus number/weight of fostered piglets in). The number of suckling piglets per sow (total number of piglets born alive plus or minus the number of piglets fostered in or out of the litter; PigRisk) and the piglets’ mortality were defined at day 10, calculated as the number of dead piglets divided by PigRisk. Table 1 reports the recorded data of sow productive traits by parity. Colostrum was collected immediately before or at the beginning of the natural farrowing, without the use of oxytocin, by using hand pressure exerted approximately in the center of the mammary gland. A total of 30 mL of colostrum was collected from each sow by pooling the fluid collected from each functional teat located in the upper row and lower row of the udder, when possible without disturbing the sow. Samples were drawn, frozen, and stored at −80 °C until further analysis.

### 2.2. Colostrum Preparation for ^1^H-NMR Analysis

Colostrum was prepared as reported by Picone et al. [17]. Briefly, colostrum was thawed and mixed by inversion; then, 15 mL of each sample was diluted 1:1 with pure water and 0.02% of sodium azide added to inhibit bacterial growth. The diluted sample was defatted through three consecutive centrifugations at 4 °C for 30 min at 1500× *g*. After each centrifugation, the aqueous phase was transferred to a clean Falcon tube, avoiding the outer layer of fat, and well mixed. Five milliliters of the obtained aqueous phase were then filtered by centrifugation at room temperature for 90 min at 5500× *g* through Amicon Ultra 10-kDa membrane centrifugal filters (Merck Millipore, Merck KGaA, Darmstadt, Germany) to eliminate high-molecular-weight proteins and immunoglobulins. The eluted samples were then immediately frozen and stored at −80 °C until ^1^H-NMR analysis. Before acquiring, 540 μL of eluted sample was centrifuged at 14,000× *g* for 15 min (Scilogex D3024 High Speed MicroCentrifuge, Rocky Hill, CT, USA) and then added to 720 μL of distilled water and 100 μL of a D_2_O solution of 3-(trimethylsilyl)-propioniate-2,2,3,3-d4 (TMSP) (Cambridge Isotope Laboratories Inc., Tewksbury, MA, USA) with a final concentration of 6.25 mmol/L. Finally, 650 μL of mixed preparation was transferred to a 5-mm glass tube for ^1^H-NMR.

### 2.3. ^1^H-NMR Measurements

^1^H-NMR spectra were recorded at 298 K with an AVANCE spectrometer (Bruker BioSpin, Karlsruhe, Germany) operating at a frequency of 600.13 MHz, equipped with an autosampler with 60 holders. The deuterated water (HOD) residual signal was suppressed by applying the NOESYGPPR1D sequence (a standard pulse sequence included in the Bruker library) incorporating the first increment of the NOESY pulse sequence and a spoil gradient. Each spectrum was acquired using 32 K data points over a 7211.54 Hz spectral width (12 ppm) and adding 256 transients. A recycle delay of 5 s and a 90° pulse of 11.4 μs were set up. Acquisition time (2.27 s) and recycle delay were adjusted to be five times longer than the longitudinal relaxation time of the protons under investigation, which was no longer than 1.4 s. The data were Fourier-transformed and phase and baseline corrections were automatically performed using TopSpin software, version 3.0 (Bruker BioSpin, Karlsruhe, Germany). Signals were assigned through a combination of literature assignments and using a multimedia library included in Chenomx NMR Suite 8.2 professional software (Chenomx, Edmonton, Alberta, Canada). Chenomx NMR Suite version 8.2 includes a metabolite library constructed by chemically modeling compounds of interest using their peak center and J-coupling information. It was used also to quantify metabolites amount based on data in an NMR spectrum with a great signal deconvolution routine. The internal standard TMSP was used as a chemical shift reference set at 0.0 ppm and to determine the amounts of the metabolites. The matching was led with a fitting procedure reproducing metabolite line shapes according to a very extended library.

### 2.4. Colostrum ^1^H-NMR Spectrum and Assigned Metabolites

Each ^1^H–NMR spectrum was processed by means of scripts in R (version 3.6) (REF) language developed in-house as follows [17]: spectrum baseline was adjusted by employing the signal identification algorithm named “baseline.peakDetection” from R package “Baseline” [19]. Chemical shift referencing was performed by setting the TMSP signal to 0.00 ppm. Spectral regions including only noise (the spectrum edges between 8.70 and 11.00 and between −0.15 and 0.15) and signals strongly affected by the residual solvent signals (water, between 4.70 and 5.10 ppm) were removed prior to data analysis. The region spectra were then normalized by means of the probabilistic quotient normalization method (PQN) [20] and divided into 204 bins of 0.0402 ppm each. Data of detected signals are reported in Table S2 (Supplementary Materials).

A total of 33 compounds listed in Table 2 were identified through a combination of literature assignments [17,21] and using a multimedia library included in Chenomx NMR Suite 8.2 professional software (Chenomx, Edmonton, AB, Canada).

### 2.5. Immunoglobulin Analysis

Samples were prepared and analyzed as reported by Balzani et al. [22]. Briefly, colostrum was thawed and mixed by inversion, and a drop of well-mixed whole colostrum was placed on a Brix refractometer (MA871 digital; Obione, La Valette, France) prism, where the Brix percentage (%) was recorded. The Brix refractometer was calibrated with distilled water before each set of analyses.

### 2.6. Statistical and Bioinformatics Analyses

The processed spectra underwent multivariate statistical analysis; in particular, principal component analysis (PCA) was applied to identify possible outliers and evaluate the PA effect. PCA was performed on the total bins (204) and 94 samples. Samples were then visualized in an individual score plot. Furthermore, 33 metabolites were then assigned to the most discriminant bins chosen to have a loading value greater than 1% of the overall standard deviation of all loading values. A second PCA was then carried out on the dataset composed by the identified metabolites in order to evaluate the effect of PA on the known compounds, and an additional PCA was performed using the matrix composed only by not assigned bins (130) and 94 samples in order to evidence the variances explained by not identified compounds. The “plotVar” function was then used to identify the structure of the correlation between binned spectra or assigned metabolites and PA. Furthermore, in order to disclose the effect of PA on the identified metabolites, an ANOVA model and Tukey’s honest significance test was carried out at a 95% confidence level (*p* ≤ 0.05). Results were considered significant at *p* ≤ 0.05 and tendencies at 0.05 ≤ *p* ≤ 0.10.

Statistical analyses on spectral data were performed using R computational language (ver. 3.6.0) [23]. The package “Mixomics” [24] was used to perform the PCA analysis, the packages “car” [25], “lsmeans” [26], and “multcomp” [27] within R software were used to compute the ANOVA and Tukey test analysis.

#### Association between Productive Traits, Parity, and Assigned Metabolites

Stepwise regression analysis was used to select, among the assigned metabolites and sows’ reproductive performance, the variables that influenced the Brix percentage estimate of IgG concentration, littersizeBA0, littersizeTB0, littersizeBA10, LitterWe0, LitterWe10, PigRisk, and piglet mortality. The results obtained from the stepwise regression analysis were then confirmed through an ANOVA model in which PA was added as additional fixed factor. The following contrasts were tested to compare the different PA: PA1 vs. PA2, PA1 vs. PA3, PA1 vs. PA4, PA2 vs. PA3, PA2 vs. PA4, and PA3 vs. PA4.

The ANOVA analysis and the stepwise regression analysis were carried out on R software using “mass” and “car” packages [25,28].

## 3. Results

### 3.1. Parity Effect on Colostrum Composition

**Figure 1:**
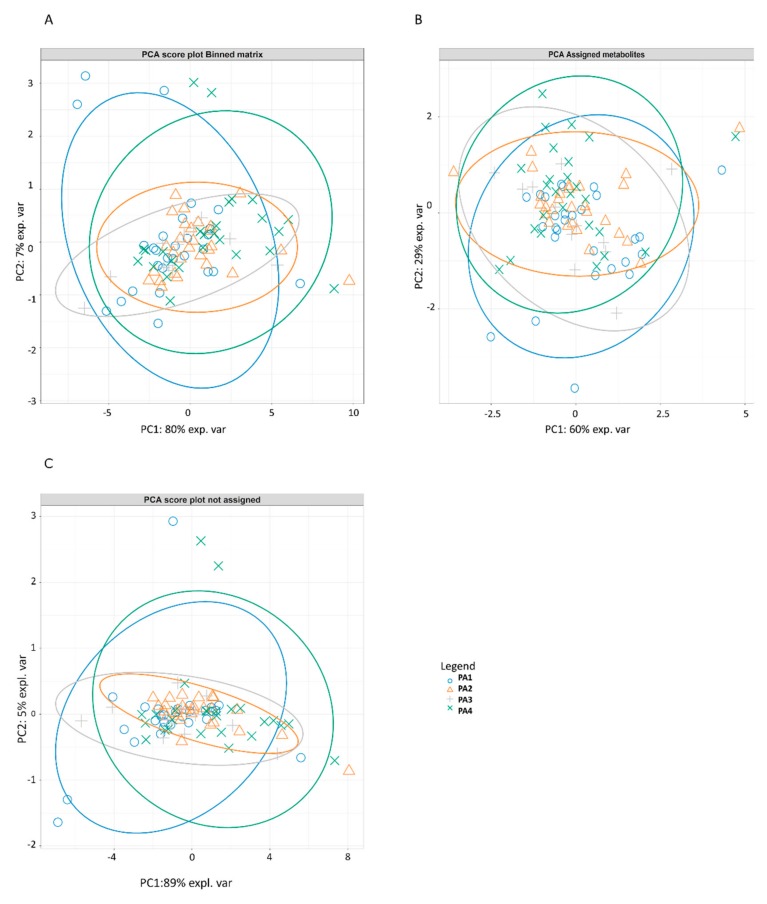
Individual score plot of principal component analysis (PCA) on the total binned spectra matrix (**A**), the assigned compounds (**B**), and the un-assigned binned spectra matrix (**C**). PCA score plot showing principal components 1 and 2 (PC1 and PC2). Shape refers to the respective sow’s parity.

The results of the PCA analysis on the total binned spectra matrix, on the matrix composed of the assigned metabolites, and on the matrix composed only by un-assigned binned spectra are reported in Figure 1A–C, respectively. No outlier was identified among samples. All parity groups partially overlapped in all the PCAs, impairing clustering recognition. For the PCA performed on the total binned matrix, the total explained variance was 10.77; the first principal component (PC1) explained 80% of the total variance and partially separated the colostrum spectra of PA1 to PA4 groups, while PC2 explained 7% of the colostrum spectra variance. The most correlated binned spectra (loadings) were signals overlapping in the midfield region between 3.310 and 4.679 ppm (Table 3). For the PCA performed on the assigned metabolites matrix, the total explained variance was 3.20; PC1 and PC2 explained 54% and 32%, respectively, of the total variance observed in the colostrum. The most correlated loadings were assigned to lactose, glycine, *O*-acetylcholine, *O*-phosphocholine, *sn*-glycero-3-phosphocholine, and choline (Table 4). For the PCA performed on the un-assigned binned spectra matrix, the total explained variance was 6.99; PC1 and PC2 explained 89% and 4.6% of the total variance and partially separated the PA1 and PA4 groups. The most correlated binned spectra (loadings) were signals overlapping in the midfield region between 3.713 and 3.874 ppm (Table 5).

**Figure 2:**
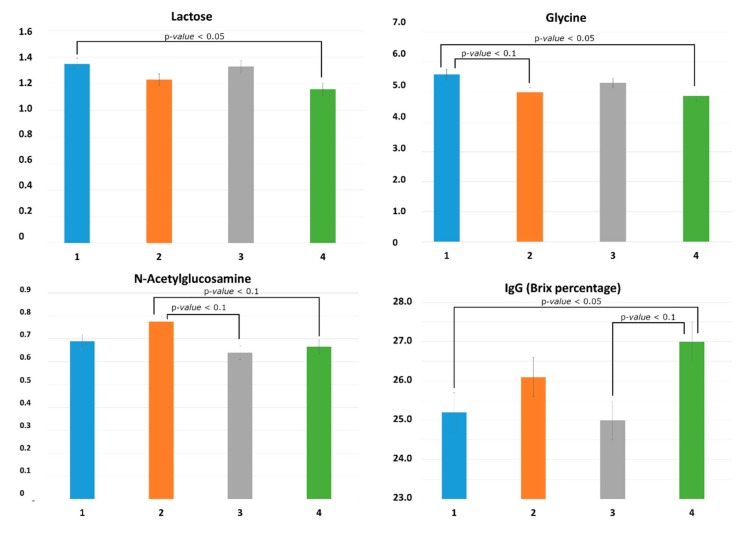
Effect of sow parity (PA) on metabolites and Brix percentage estimate of immunoglobulin G (IgG) in colostrum. Metabolite concentrations were considered in area arbitrary units; IgG concentration is expressed in Brix percentage. Lactose: PA1 vs. PA4, *p* < 0.05; glycine: PA1 vs. PA4, *p* < 0.05; PA1 vs. PA2, *p* < 0.1; *N*-acetylglucosamine: PA2 vs. PA3, *p* < 0.1; PA2 vs. PA4, *p* < 0.1; IgG: PA1 vs. PA4, *p* < 0.05; PA3 vs. PA4, *p* < 0.1.

The univariate approach (ANOVA coupled with the contrasts) showed that PA influenced the quantity of lactose (*p* = 0.027), glycine (*p* = 0.026), and *N*-acetylglucosamine (GlcNAc, *p* = 0.040) (Table S3, Supplementary Materials). In detail, lactose and glycine were higher in PA1 compared with PA4 (mean ± standard error (SE) reported in area arbitrary units; lactose: PA1 = 1.35 ± 0.04, PA4 = 1.16 ± 0.05; *p* = 0.028; glycine: PA1 = 5.59 ± 0.18, PA4 = 4.87 ± 0.18; *p* = 0.028), while a trend for higher quantity was observed in PA1 compared to PA2 for glycine (PA1 = 5.59 ± 0.18, PA2 = 4.99 ± 0.18; *p* = 0.080) and a trend for higher GlcNAc was observed comparing PA2 to both PA3 and PA4 (PA2 = 0.77 ± 0.03; PA3 = 0.64 ± 0.04; PA4 = 0.67 ± 0.03; PA2 vs. PA3 P = 0.081; PA2 vs. PA4; *p* = 0.071) (Figure 2).

PA tended to influence the Brix percentage estimate of IgG concentration (*p* = 0.10); the PA4 (Brix% 27 ± 0.57) had a significantly higher Brix percentage estimate of IgG concentration compared to PA1 (25.2 ± 0.55; *p* = 0.028) and tended to have a higher Brix percentage compared with PA3 (25.0 ± 0.85; *p* = 0.059) (Figure 2).

### 3.2. Association between Metabolites and IgG in Swine Colostrum

The stepwise regression analysis revealed that specific metabolites were associated with Brix percentage estimate of IgG concentration in colostrum. The quantity of lactose was negatively associated with the Brix percentage estimate of IgG concentration, while the quantity of creatine, *myo*-inositol, and *O*-phosphocholine were positively associated with the Brix percentage estimate of IgG concentration (Table 6). The association between these metabolites and Brix percentage estimate of IgG was then tested using an ANOVA model and, for all the metabolites, the effect was confirmed (Table 7) (*p* < 0.005). PA of the sow was also included in the ANOVA model, but it did not significantly influence the Brix percentage estimate of IgG concentration.

### 3.3. Association between Colostrum Metabolites and Sow Performance Traits

Colostrum composition and PA of the sow were not related to the littersizeTB0, littersizeBA0, littersizeBA10, livedead, stillborn, PigRisk, and LitterWe10. The stepwise regression analysis revealed that, in addition to the influence of littersizeBA0, the concentration of taurine was associated with the LitterWe0 (Table 6). The association between taurine and LitterWe0 (*p* < 0.0001) was then tested and confirmed using an ANOVA model, showing that taurine concentration was positively associated (coefficient: 16.26) with the LitterWe0 (Table 7). Piglet mortality was associated with the quantity of GlcNAc and of *myo*-inositol using the stepwise regression analysis (Table 6). The association between GlcNAc (coefficient: −0.43) and *myo*-inositol (coefficient: 0.38) and the piglet mortality was then confirmed using an ANOVA model (*p* < 0.05) (Table 7). In addition, piglet mortality was significantly associated with the LitterWe10 (coefficient = −0.01; *p* = 0.005).

## 4. Discussion

In the present study, the ^1^H-NMR technique allowed the identification of a total of 33 compounds, some of which were previously identified in swine colostrum using the same technique [17]. In addition to the compounds identified by Picone et al. [17], in the present study, 12 new compounds were identified, including several amino acids (glycine, leucine, threonine, tyrosine, valine, and phenylalanine), nucleotides (uracil), nucleoside (inosine), organic acid (*trans*-aconitate), and additional functional compounds such as betaine, *O*-phosphocholine, and *O*-acetylcarnitine, while glycolate, *N*-acetylglutamate, succinate, and uridine diphosphate (UDP)-glucose were not identified. Differences in the detected compounds may be due to the difference in the animals’ health status, in the animals’ diet and management, and in animal genetics among studies. The ^1^H-NMR technique represents one of the most sensitive techniques that allows detection of metabolites in bio-fluids with high sensitivity, and it is quantitative, highly reproducible, and straightforward to use [29,30]. The present study investigated if the parity of the sow can influence the metabolomics profile of swine defatted colostrum and explain differences in the productive performance.

The characterization of the colostrum metabolomics profile showed that colostrum soluble compounds were only slightly affected by the sow’s parity number; indeed, poorly separated clusters due to parity number were observed using the PCA analysis, both in the whole metabolomics matrix (identified and unidentified compounds) and in the matrix composed only of identified compounds. The results of the PCAs report that, although parity is not a factor capable of strongly influencing the composition of the defatted colostrum, a part of the spectrum located in the midfield region between 3.713 and 3.874 ppm, which explains 6% of the variance, still remains to be investigated for the identification of compounds distinguishing the colostrum matrix.

Although the parity did not strongly influence the metabolomics profile of colostrum, it significantly influenced the quantity of some compounds including lactose, glycine, and GlcNAc. Lactose quantity is generally considered stable in colostrum and milk. Previous studies did not detect differences in lactose amount among different parities [11,31]. In the present study, the amount of lactose was lower in the fourth-parity sows compared to gilts, which is consistent with a previous report in mature milk [11]. Lactose is generally associated with the total amount of produced and suckled milk, as it is the principal osmotic compound in the mammary secretions [32] and its synthesis by mammary epithelial cells is known to be stimulated by prolactin and inhibited by progesterone. Previous studies suggested that colostrum yield can vary across parity [33,34]. However, in the present study, colostrum yield was not measured; thus, the difference in lactose content among parities cannot be directly associated to colostrum quantity, and this hypothesis should be further investigated. Similarly to lactose, glycine content was also higher in gilts’ colostrum compared to fourth-parity sows. Glycine was recently related to a negative maternal energy balance in cows [35]. In the present study, the sows’ energy balance was not assessed; however, it is well known that gilts have a more negative energy balance compared to multiparous sows [36,37], which may explain the higher level of glycine in gilt colostrum.

As expected, the colostrum of gilts had a lower Brix percentage estimate of IgG concentration compared with fourth-parity sows, which is consistent with previous reports [12,38]. However, the variability in Brix percentage estimate of IgG explained by the sows’ parity disappeared when the statistical model was fitted including the metabolites. In fact, the Brix percentage estimate of IgG was negatively associated with the level of lactose and positively associated with the level of *myo*-inositol, creatine, and *O*-phosphocholine. No previous studies investigated the relationship between IgG and other metabolite concentrations in swine colostrum, and scarce information on those metabolites is available.

Lactose is the principal osmotic compound in colostrum and milk. Since a higher milk/colostrum yield was associated with a higher lactose content [34], it may result in a dilution of the Brix percentage estimate of IgG concentration in colostrum observed in the present study. However, since no information in colostrum yield is available in the present study, further investigation should be made to confirm the hypothesis. In addition, it was reported that the level of lactose in colostrum/milk negatively influenced the piglets’ absorption of IgG and favored gut closure, which occurs within 24–36 h of birth [39]. The discussed hypothesis cannot be assessed in the present study and does not explain the negative relationship between lactose and Brix percentage estimate of IgG. Thus, further investigations are needed to clarify the IgG and lactose relationship in swine colostrum.

In contrast, creatine, *myo*-inositol, and *O*-phosphocholine were positively correlated with the Brix percentage estimate of IgG. No previous information was reported on the relationship between these compounds and the IgG content; nevertheless, beneficial proprieties were attributed to these compounds. The presence of creatine in swine colostrum and milk, as well as its importance for newborn development and maturation, is well documented [17,40,41,42]. Furthermore, it is known that creatine kinase, an enzyme that catalyzes the production of creatine, can create a macromolecular complex with IgG (IgG-CK) [43], which may explain the association between IgG percentage and creatine amount observed in the present study; however, further elucidation is needed.

*O*-Phosphocholine, as a form of storage for choline, is considered a precursor for the biosynthesis of phospholipids (phosphatidylcholine and sphingomyelin), which are essential structural components of all cellular membranes [44]. *myo*-Inositol, a glucose derivative generally known also as inositol, is widely used as a nutritional supplement in human preterm infants for its critical role in fetal and early neonatal life, especially for lung development [45]. Furthermore, a genetic effect was seen for creatine and *myo*-inositol in sow colostrum, with Duroc sows having lower values than Landrace and Large White sows [17].

In the present study, metabolites identified in colostrum were associated with sow performance at farrowing and 10 days post farrowing. Although the effect of sow parity and colostrum composition on piglet performance at 10 days should be interpreted with caution due to the intense cross-fostering, the *myo*-inositol in colostrum was positively associated with piglet mortality at day 10 which was not expected considering the positive properties that this compound has during neonatal life. However, since a higher level of *myo*-inositol was found in human colostrum of pre-term parturition compared to normal parturition [46], this may suggest that the increased *myo*-inositol secretion in colostrum might reflect the physiological state and the immaturity of the mammary gland and the neonate immaturity, which would indirectly explain the positive association between *myo*-inositol and the mortality observed in our study. In humans, the *myo*-inositol quantity decreases from colostrum to milk [46], while no information was reported in colostrum/milk transition of sows. Thus, further studies on *myo*-inositol concentration across lactation are needed, to also confirm the association between *myo*-inositol and piglet mortality.

Piglet mortality at day 10 was reduced by increasing levels of colostrum GlcNAc. Information on the role that the amino sugar GlcNAc plays in neonates is scarce; however, its presence in colostrum and milk was previously reported [17,47]. It is known that GlcNAc has important structural roles at the cell surface level (i.e., bacterial cells, fungal cells, and the extracellular matrix of animal cells) and it also exerts cell signaling activities [48]. These properties may explain its positive effect in reducing piglet mortality; however, further studies to explain these mechanisms more thoroughly are needed.

The litter weight at birth was affected by the litter size, which was an expected result, but it was also positively associated with the taurine concentration in colostrum. Taurine is one of the main free amino acids in colostrum, and it is known to improve newborn brain development [49]. In the study of Picone et al. [17], taurine concentration in colostrum was associated with a lower number of dead piglets at three days; in the present study, mortality was not recorded at three days, and taurine did not affect the mortality at 10 days; thus, results cannot be compared. The result for taurine observed in the present study should be related to the metabolism of the sow rather than to the function of taurine in the neonates. Taurine in colostrum is derived from the diet or can be biosynthesized from methionine and cysteine metabolism in the liver and in other tissues, including the oviduct epithelial cells [50]. Our results suggest that sows having colostrum richer in taurine also had a heavier litter at birth, resulting in better productive traits, and this may reflect a better health status of the sow. Further elucidations on the role that the identified compounds have in piglets’ metabolism and health are desirable. Furthermore, the relevance of sow metabolic condition for colostrum quality is also indicated by the association of backfat of the sow at farrowing with the concentration of IgA in colostrum, when equalized for the parity number of the sow [51]. Thus, the relationship between sows’ energy balance and colostrum metabolomics composition deserves further investigations in order to improve sows’ management and, consequently, the colostrum quality.

## 5. Conclusions

In relation to the hypothesis of the study, the results indicate that the metabolomics composition of swine defatted colostrum is only slightly influenced by parity, which influenced the quantity of a limited number of assigned metabolites including glycine and lactose. From the results obtained, our study suggests that specific metabolites in colostrum can partially explain the variability in colostrum Brix percentage estimate of IgG concentration and of sows’ productive traits, including litter weight at birth and piglets’ mortality. Although further studies that allow control of additional factors including cross-fostering, calculation of colostrum yield, and sire effects are needed to fully characterize the metabolites composition of colostrum and the association with performance and survival of piglets, these results are promising in order to improve colostrum quality. Moreover, a better understanding of the influential metabolites in colostrum will allow scientists to modify management and nutrition strategies to provide weak piglets with good-quality colostrum, improving welfare and economy by reducing piglet mortality.

## Figures and Tables

**Table 1:** Descriptive statistics of sow production traits by parity number.

Item ^1^	Parity Number							
	1		2		3		4	
	Mean	SD	Mean	SD	Mean	SD	Mean	SD
LittersizeBA0	11.79	2.08	12.79	2.06	13.00	2.63	13.00	2.30
LittersizeTB0	12.32	2.31	13.29	2.40	13.83	2.76	13.92	2.04
LitterWe0	16.50	2.34	18.24	3.32	17.44	4.04	18.34	3.24
LitterWe10	39.89	9.18	43.45	7.54	42.78	8.20	40.90	7.14
Livedead	0.64	1.52	0.39	0.74	1.08	1.51	1.00	1.47
Stillborn	0.54	0.69	0.50	0.79	0.83	0.94	0.92	1.16
PigRisk	11.50	1.48	12.25	1.78	12.50	1.93	12.38	1.92
Mortality	0.08	0.17	0.04	0.07	0.09	0.11	0.08	0.10
IgG (Brix %)	25.17	2.40	26.13	3.12	25.00	2.23	26.95	3.46

^1^ LittersizeBA0: total number of piglets born alive; LittersizeTB0: total number of piglets born; LitterWe0: litter weight at birth, excluding stillborns; LitterWe10: litter weight at day 10, excluding dead piglets; livedead: number of dead piglets in each litter at day 10; stillborn: number of stillborn piglets; PigRisk: number of suckling piglets per litter after fostering; Mortality: number of dead piglets divided by PigRisk at day 10; IgG: immunoglobulin G (Brix %).

**Table 2:** Assignment table of the identified metabolites present in the ^1^H-NMR spectra of swine colostrum.

Assigned Number	^1^H Chemical Shift (ppm) ^1^	Compound
1	0.994 (d)	Valine
2	0.969 (t)	Leucine
3	1.333 (d)	Threonine
4	1.334 (d)	Lactate
5	1.485 (d)	Alanine
6	1.925 (s)	Acetate
7	2.003–2.083 (s)	*N*-Acetylglucosamine
8	2.534 (d)–2.66 (d)	Citrate
9	2.645 (d)–3.197 (s)	*O*-Acetylcarnitine
10	2.716 (s)	Dimethylamine
11	3.039 (s)	Creatine
12	3.047 (s)	Creatine phosphate
13	3.051 (s)	Creatinine
14	3.197 (s)	Choline
15	3.206 (s)	*O*-Phosphocholine
16	3.223 (s)	*O*-Acetylcholine
17	3.231 (s)–4.330 (m)	*sn*-Glycero-3-phosphocholine
18	3.290 (t)–3.539:3.956 (m)–4.447 (d)–4.668 (d)–5.231 (d)	Lactose
19	3.269 (s)	Betaine
20	3.276 (t)–3.425 (t)	Taurine
21	3.540 (s)	Glycine
22	4.078 (t)	*myo*-Inositol
23	5.607 (dd)–7.946 (d)	UDP-Galactose
24	5.692 (m)	*cis*-Aconitate
25	5.811 (d)–7.557 (d)	Uracil
26	5.912 (d)–5.924 (d)–7.946	UDP-*N*-Acetylglucosamine
27	6.105 (d)–8.210 (s)–8.358 (s)	Inosine
28	6.591 (s)	*trans*-Aconitate
29	6.910 (m)–7.198 (m)	Tyrosine
30	7.336 (d)–7.379 (m)–7.437 (m)	Phenylalanine
31	7.882 (d)	Uridine
32	8.106 (d)	UMP
33	8.461 (s)	Formiate

^1^ The assignments were obtained at pH 7.24. Chemical shift values are referenced to 3-(trimethylsilyl)-propioniate-2,2,3,3-d4 (TMSP) proton signals at 0.00 ppm. (d, doublet; dd, doublet of doublets; m, multiplet; s, singlet; t, triplet). UDP—uridine diphosphate; UMP—uridine monophosphate.

**Table 3:** Most correlated bins of swine colostrum obtained from the principal component analysis (PCA) carried out on the binned matrix.

ppm	PC1 ^1^	PC2 ^2^	Assigned Compound
4.679	−0.85	−0.33	Lactose
4.478	−0.81	−0.56	UMP
3.955	−0.93	−0.18	Lactose
3.915	−0.83	0.16	Not assigned
3.874	−0.93	−0.03	Not assigned
3.834	−0.93	−0.32	Not assigned
3.794	−0.99	−0.09	Not assigned
3.754	−0.90	0.00	Not assigned
3.713	−0.93	−0.06	Not assigned
3.673	−0.97	0.03	Lactose
3.633	−0.87	0.43	Lactose
3.593	−0.87	0.37	Lactose
3.552	−0.93	0.31	Glycine
3.311	−0.89	−0.41	Glycine

^1^ Principal component 1; ^2^ principal component 2.

**Table 4:** Most correlated metabolites of swine colostrum obtained from the principal component analysis carried out on the assigned metabolite matrix.

Metabolite	ppm	PC1 ^1^	PC2 ^2^
Lactose	4.40; 5.207	−0.23	0.83
Glycine	3.54	−0.45	0.87
*O*-acetylcholine	3.223	−0.95	−0.30
*O*-phosphocholine	3.206	−0.92	−0.33
*sn*-glycero-3-phosphocholine	3.232	−0.97	−0.15
Choline	3.19	−0.95	−0.30

^1^ Principal component 1; ^2^ principal component 2.

**Table 5:** Most correlated un-assigned bins of swine colostrum obtained from the principal component analysis carried out on the un-assigned binned matrix.

ppm	PC1 ^1^	PC2 ^2^
3.713	−0.91	0.34
3.754	−0.87	0.45
3.794	−0.99	−0.08
3.834	−0.91	0.04
3.874	−0.91	0.32

^1^ Principal component 1; ^2^ principal component 2.

**Table 6:** Results of stepwise analysis on sows’ productive traits.

Variable	Estimate	SE	T-Value	*p* (>|t|)
Model for IgG: *R*^2^ = 0.55, *R*^2^ (adjusted) = 0.53, Cp = 74.41				
(Intercept)	23.95	1.52	15.74	<0.0001
Lactose ^1^	−8.89	1.02	−8.70	<0.0001
Creatine ^1^	27.26	5.16	5.28	<0.0001
*myo*-Inositol ^1^	5.58	1.75	3.19	0.002
*O*-Phosphocholine ^1^	1.18	0.40	2.96	0.004
Model for mortality ^2^: *R*^2^ = 0.15, *R*^2^ (adjusted) = 0.127017, Cp = −1.92				
(Intercept)	0.04	0.08	0.54	0.590
*N*-Acetylglucosamine ^1^	−0.46	0.13	−3.61	0.001
*myo*-Inositol ^1^	0.40	0.13	2.96	0.004
Model for LitterWe0 ^3^: *R*^2^ = 0.75, *R*^2^ (adjusted) = 0.748687, Cp = −2.44				
(Intercept)	−0.39	1.20	−0.33	0.743
LittersizeBA0 ^4^	1.15	0.08	15.27	<0.0001
Taurine ^1^	16.26	4.11	3.96	<0.0001

^1^ Metabolite concentrations were considered in area arbitrary units; ^2^ mortality^:^ number of dead piglets divided by PigRisk at day 10; ^3^ LitterWe0: litter weight at birth; ^4^ LittersizeBA0: total number of piglets born alive. SE—standard error.

**Table 7:** Results of ANOVA analyses on Brix percentage estimate of colostrum immunoglobulin G concentration and sows’ productive traits.

Variable	Df	Sum of Squares	Coefficient	F-Value	*p* (>F)
Model for IgG					
Lactose ^1^	1	315.10	−8.89	75.64	<0.0001
Creatine ^1^	1	116.36	27.26	27.93	<0.0001
*myo*-Inositol ^1^	1	42.30	5.58	10.15	0.002
*O*-Phosphocholine ^1^	1	36.50	1.18	8.76	0.004
Residuals	86	362.40			
Model for mortality ^2^					
*N*-Acetylglucosamine ^1^	1	0.14	−0.43	12.56	0.001
*myo*-Inositol ^1^	1	0.10	0.38	8.73	0.004
LitterWe10 ^3^	1	0.09	−0.01	8.20	0.005
Residuals	74	0.84			
Model for LitterWe0 ^4^					
LittersizeBA0 ^5^	1	596.79	1.15	233.04	<0.0001
Taurine ^1^	1	40.17	16.26	15.69	<0.0001
Residuals	91	233.05			

^1^ Metabolite concentrations were considered in area arbitrary units; ^2^ mortality: number of dead piglets divided by PigRisk at day 10; ^3^ LitterWe10: litter weight at day 10, excluding dead piglets; ^4^ LitterWe0: litter weight at birth; ^5^ LittersizeBA0: total number of piglets born alive. Df—degrees of freedom.

## Supplementary Materials

The following are available online at https://www.mdpi.com/2076-2615/10/1/154/s1, Table S1. Ingredients and composition of the sows’ diet expressed as fed, Table S2. Data of detected signals in swine defatted and ultrafiltered colostrum. Table S3. Effect of sow parity on the assigned metabolites in the defatted and ultra filtered colostrum.
